# A Review of Recent Curcumin Analogues and Their Antioxidant, Anti-Inflammatory, and Anticancer Activities

**DOI:** 10.3390/antiox13091092

**Published:** 2024-09-06

**Authors:** Kirandeep Kaur, Ahmad K. Al-Khazaleh, Deep Jyoti Bhuyan, Feng Li, Chun Guang Li

**Affiliations:** 1 NICM Health Research Institute, Western Sydney University, Penrith, NSW 2751, Australia; 19316068@student.westernsydney.edu.au (A.K.A.-K.); d.bhuyan@westernsydney.edu.au (D.J.B.); 2 School of Science, Western Sydney University, Parramatta, NSW 2150, Australia; feng.li@westernsydney.edu.au

**Keywords:** curcumin, anti-inflammatory, antioxidant, anticancer, curcumin analogues

## Abstract

Curcumin, as the main active component of turmeric (*Curcuma longa*), has been demonstrated with various bioactivities. However, its potential therapeutic applications are hindered by challenges such as poor solubility and bioavailability, rapid metabolism, and pan-assay interference properties. Recent advancements have aimed to overcome these limitations by developing novel curcumin analogues and modifications. This brief review critically assesses recent studies on synthesising different curcumin analogues, including metal complexes, nano particulates, and other curcumin derivatives, focused on the antioxidant, anti-inflammatory, and anticancer effects of curcumin and its modified analogues. Exploring innovative curcumin derivatives offers promising strategies to address the challenges associated with its bioavailability and efficacy and valuable insights for future research directions.

## 1. Introduction

Turmeric is one of the most used culinary spices in Asian countries. Turmeric is the dried rhizome of *Curcuma longa* L. and is cultivated in tropical and subtropical regions. India is the world’s largest producer and consumer of turmeric. The rich yellow colour and potential biological activities of turmeric are attributed to the presence of curcuminoids [1]. Turmeric has been traditionally used as a medicinal herb to promote blood and relieve pain in Asian countries (mainly India and China) [1]. Curcumin, a polyphenol, is found to be the major bioactive compound in turmeric.

As a PAINS (pan-assay interference) compound, curcumin demonstrates various types of behaviours. It exhibits covalent labelling of proteins, metal chelation, redox reactivity, aggregation, membrane disruption, fluorescence interference, and structural decomposition. As a result, when determining the activity of curcumin using different assays, these potential modes of assay interferences need to be accounted for [2]. In the global literature, two major red flags have popped up on the bioactivity profiles of curcumin reported to date: (1) the rate at which this compound, or mixture, is reported as being bioactive and, especially, (2) the relatively high ratio of positive activities seen in proportion to the total number of distinct bioactivities reported: just over 300, as assessed using the NAPRALERT database [2].

**Figure 1:**
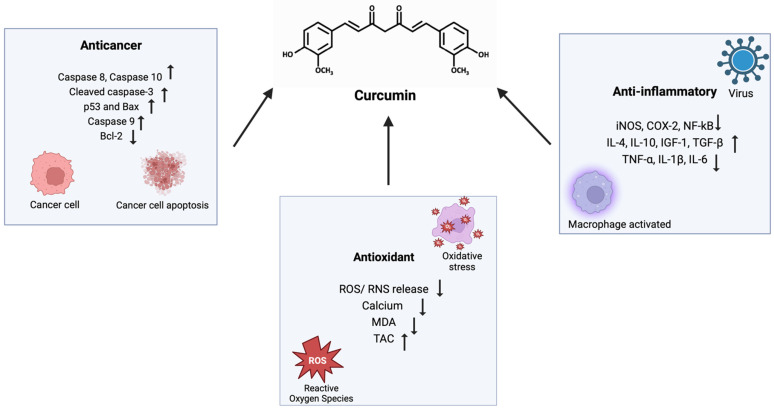
Major biological properties and pathways of curcumin.

Curcumin has been shown to exhibit antioxidant, anti-inflammatory, antimicrobial, anticancer, and antimutagenic properties, enabling it to be used as a supplement for various health conditions. Several reports over the last few decades have specified the potent therapeutic potential of curcumin against various cancers [3]. It has been shown to prevent the growth and metastasis of various tumours through the regulation of different transcription factors, growth factors, inflammatory cytokines, protein kinases, and enzymes [3]. Some of the key pathways of curcumin, as per their biological properties, are highlighted in Figure 1. Curcumin also inhibits the proliferation of cancer cells, induces apoptosis, and suppresses angiogenesis [4]. This review provides an overview of some of the novel curcumin analogues, metal complexes, nano particulates, and various curcumin derivatives that have shown promising and enhanced anticancer, anti-inflammatory, and antioxidant activities when compared to curcumin. Furthermore, we discuss the potential clinical benefits of these curcumin modifications in the treatment of various diseases while proposing several exciting directions for future research.

## 2. Chemistry of Curcumin—Structure and Properties

Curcumin (diferuloylmethane), an orange-yellow crystalline powder (molecular formula of C_21_H_20_O_6_), is an active compound of the perennial herb *Curcuma longa* L. (commonly known as turmeric). The yellow-pigmented fraction of *Curcuma longa* contains curcuminoids (demethoxycurcumin and Bis-demethoxycurcumin), which are chemically related to its principal ingredient, curcumin. It was first isolated in 1815 by Vogel and Pelletier, obtained in crystalline form in 1870, and identified as 1,6-heptadiene-3,5-dione-1,7-bis(4-hydroxy-3-methoxyphenyl)-(1E,6E) or diferuloylmethane [5]. The feruloyl methane structure of curcumin was subsequently confirmed in 1910 through the original work and synthesised by Lampe [6]. Curcumin is very little or not soluble at all in aqueous solutions. Still, it is soluble in organic solvents such as dimethyl sulfoxide (DMSO), ethanol, methanol, or acetone. It has a melting point of 183 °C and molecular weight of 368.37 g/mol [6,7,8].

**Figure 2:**
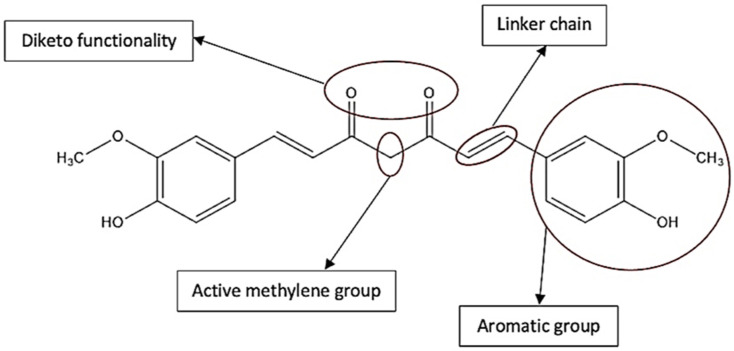
Curcumin structure shows four major reactive sites.

Spectrophotometrically, curcumin has a maximum absorption (λ_max_) of 430 nm in methanol. In acetone, the maximum absorbance of curcumin can be accomplished at 415 to 420 nm [8]. Curcumin gives a bright yellow hue at a pH of 2.5 to 7 and changes to red when it reaches a pH of 7 (neutral) [8]. On the contrary, tetrahydrocurcumin (THC), which is one of the major metabolites of curcumin, is found to be relatively stable at neutral or basic pH. The molecule is soluble in 0.1 M sodium hydroxide (NaOH) but remains stable only for 1–2 h. A major degrading product was found to be a Trans-6-(40-hydroxy-30-methoxyphenyl)-2,4-dioxo-5-hexenal and vanillin, where ferulic acid and feruloyl methane were identified as minor degradation products. A study by Tomren demonstrated that complexation with cyclodextrin stabilises curcumin in aqueous solutions [9]. Structurally, curcumin is a symmetrical molecule consisting of four chemical entities, aryl side chains linked together by a linker in the presence of a diketo functional group, two double bonds, and an active methylene moiety (Figure 2). Studies have been performed on each of these sites in search of a potential site for suitable modifications to improve curcumin’s solubility, bioavailability, and efficacy [1]. The modification of curcumin not only improved its pharmacological activity and affected receptor binding but also enhanced its physiochemical and pharmacokinetic properties [9]. For example, several curcumin derivatives have shown enhanced antitumor and anti-inflammatory activities when compared to curcumin due to the high level of methylation, the unsaturation of the diketone moiety, and a low level of hydrogenation. In addition, many hydrogenated curcumin analogues have shown potent antioxidant activity [10].

**Figure 3:**
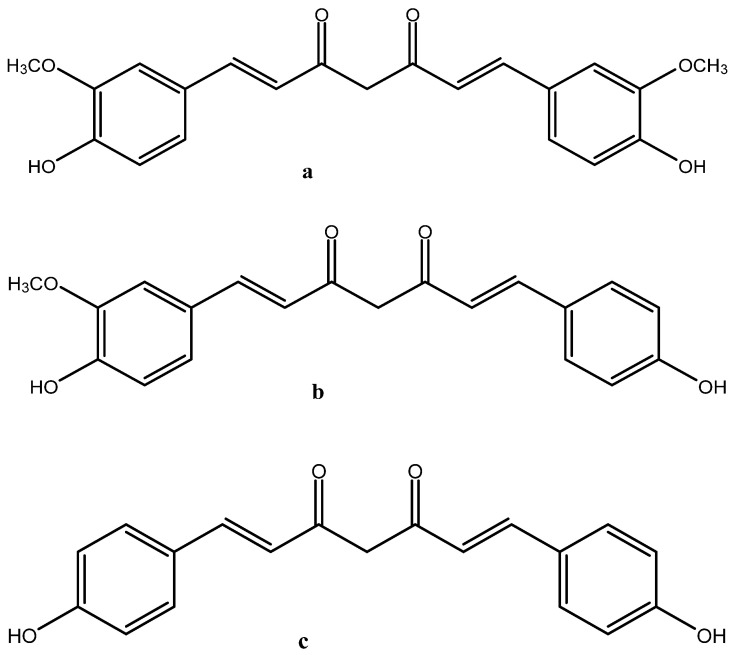
Three curcuminoids of significance: (**a**) curcumin, (**b**) demethoxycurcumin, and (**c**) bisdemethoxycurcumin.

After the work undertaken by Aggarwal and co-workers in the 1990s on its potential anticancer effect, the pace of curcumin research has grown tremendously, with more than 50,000 citations to date. Over time, curcumin has become one of the most studied topics in different branches of chemistry, including inorganic, organic, physical, and analytical chemistry. In organic and inorganic chemistry, synthetic derivatives and extraction of curcumin and metal chelating abilities through the *β*-diketo and OH groups to form novel structural entities with modified biochemical activities have been studied [11]. The perceptive difficulty of using curcumin as a potent medicinal agent is its poor solubility in an aqueous solution, which severely limits absorption and reaches optimal therapeutic activity in the human body [12]. It is reported that ethanol is the preferred solvent for extracting curcuminoids from turmeric. The natural curcuminoids of significance are curcumin (**a**), demethoxycurcumin (**b**), and bisdemethoxycurcumin (**c**) (Figure 3), which account for approximately 77%, 18%, and 5% of the composition of turmeric, respectively. Compared to other organic solvents, such as dimethyl sulfoxide (DMSO) (25 µg/mL) and ethanol (10 µg/mL), the solubility of curcumin in water (<0.1 µg/mL) is extremely low.

**Figure 4:**
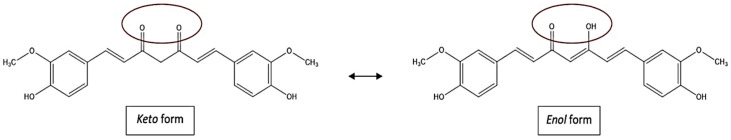
Schematic representation of keto-enol tautomerism of curcumin.

Furthermore, curcumin exists in both enolic and beta-diketone forms. It is found stable at acidic pH but unstable at neutral and basic pH, and it is then degraded down to ferulic acid and feruloyl methane [1]. A diketone moiety is formed by these carbonyl groups, which exist in keto-enolic tautomeric forms (Figure 4), where, dynamically, more stable enol-forms exist in the solid phase and in acidic solutions [13]. To yield an enolate moiety, deprotonation takes place under mild alkaline conditions. Hence, these facile tautomeric conversions are assumed to subsidise curcumin’s rapid metabolism. In unmodified curcumin, unsaturated carbonyls are a good Michael acceptor and can undergo nucleophilic additions under biological conditions that may enhance its bioavailability. Although several strategies have been tried, limited success has been achieved in terms of modulating curcumin’s metabolism, resulting in ill-defined and unstable products. As a result, several research groups have attempted and are still studying the structural motif of curcumin to slow down its metabolism and improve its potency and efficacy.

## 3. Methods

Over 130 publications and sources related to curcumin were searched to conduct literature searches, and 46 curcumin analogues studied in recent years have been reported for their chemical and biological properties, including various in vivo and in vitro properties. Relevant publications were searched in PubMed (https://pubmed.ncbi.nlm.nih.gov (accessed on 29 December 2023)), ScienceDirect (https://www.sciencedirect.com (accessed on 29 December 2023)), and Google Scholar (https://scholar.google.com (accessed on 29 December 2023)), using various names of curcumin and its related functions, such as antioxidant, anticancer, anti-inflammatory, analogues, solubility, and bioavailability as keywords. A literature search was conducted independently by the lead author (KK). The keywords were finalised by the two authors, the lead author (KK) and the corresponding author (CGL), and all the authors assessed and agreed upon them. The final list of selected publications was assessed and agreed upon by all participating authors, where overlaps were eliminated. The search was restricted to articles published only in English. The chemical structure of curcumin, its major active sites, and enol-keto tautomerism and tables were adopted with minor modifications [1].

## 4. Strategies for Improving the Therapeutic Window of Curcumin

A promising and innovative way to overcome the issue, as identified by numerous researchers, is to look for new drug delivery systems and to produce new synthetic curcumin analogues, nano particulates, and metal complexes. Curcumin modification has also facilitated overcoming drawbacks and enhanced solubility, bioavailability, and effectiveness, thus leading to higher bioactivity with reduced toxicity [14]. Curcumin has been reported to be a safe, natural therapeutic agent, as it does not cause any known severe adverse effects, even at doses as high as 8 g per day in humans [15], which might be attributed to its low solubility and bioavailability. Numerous approaches have been undertaken to improve the drug effect of curcumin. However, low water solubility and bioavailability of curcumin cause a major concern, limiting its therapeutic convenience due to the percentage (75%) of curcumin excreted in faeces, indicating its poor absorption in the gastrointestinal tract (gut) [15]. To increase the effect of curcumin, a combination of other therapeutic agents like piperine was used to interfere with glucuronidation [16]. For curcumin to be a viable therapeutic agent, two factors are considered and researched: its low water solubility and bioavailability, and the other concerns its rapid metabolism. These two factors have been tackled in different studies over the years by adopting two strategies: (1) synthesising its analogues through modification of its structural motif and (2) employing novel drug delivery systems. However, there has been limited success in avoiding curcumin’s rapid metabolism and enhancing its solubility and bioavailability even after numerous attempts [15].

## 5. Novel Drug Delivery Systems

To increase the stability of curcumin, novel drug delivery systems such as nanoparticles, metal complexes, liposomes, solid dispersion, microemulsion, micelles, nano gels, and dendrimers have been explored to increase the absorption and bioavailability of curcumin [17]. Wang and co-workers have developed curcumin micelles for the stabilisation of curcumin by a mixture of surfactant molecules, such as sodium dodecyl sulphate, cetyltrimethylammonium bromide (CTAB), Tween 80, Triton X-100, and pluronic polymers [18]. Various self-emulsifying curcumin formulations have been developed successfully with particle sizes of approximately 30 nm, and approximately 99% of curcumin loading showed a 10–14-fold greater absorption rate in male Wistar strain rats [18]. Another approach was attempted by Gao et al. (2010) for curcumin nanosuspension (CUR-NS), which was stabilised by d-*α*-tocopheryl polyethylene glycol 1000 succinate (TPGS) and was later examined for its pharmacokinetics after intravenous administration to rabbits and mice. Close observation of these formulations suggested an increase in the plasma concentration of curcumin by 3.8 times, increasing its bioavailability. In addition, micro-emulsions of curcumin, which are considered isotropic nanostructures and stable solutions comprising surfactant, oil, and water, were also prepared to increase its bioavailability [19].

Ganta and co-workers have reported that curcumin nanoemulsion when administrated orally increased the bioavailability of the standard chemotherapeutic drug paclitaxel up to 5.2-fold, and there was a 3.2-fold increase in its accumulation at the tumour site in an oral administration to SKOV-3 (human ovarian cancer) tumour-bearing xenograft mice models [20]. Nanoformulations based on dextransulfate–chitosan mixtures are widely accepted for oral, intravenous, and controlled delivery purposes. In a study by Anitha et al. (2011), quantification of the cellular uptake of curcumin encapsulated in dextransulfate–chitosan NPs was performed using the spectrophotometric method in a panel of cancer cells, including the L929 (Mouse fibroblast cells), MCF-7 (human breast cancer cells), PC-3 (human prostate cancer cells), and MG 63 (human osteosarcoma cells). The curcumin formulation showed greater inhibitory activity against the MCF-7 cells compared to the other tested cell lines [21].

Yadav and co-workers implemented this technique into their study and developed cyclodextrin–curcumin self-assembly, which exhibited higher efficacy compared to curcumin in inhibiting tumour necrosis factor (TNF)-induced expression of NF-κB regulated genes (VEGF, MMP-9, and cyclin D1) and unregulated death receptors (DR4 and DR5) in KBM-5 (myelogenous leukemia cells). As a comparison, Yallapu et al. in 2010 showed that a self-assembled curcumin complex with poly-*β*-cyclodextrin had antiproliferative properties against prostate cancer cells. The presence of curcumin in the complex downregulated antiapoptotic Bcl-2 and Bcl-xL and induced proapoptotic Bax family proteins, thus stimulating apoptosis in the prostate cancer cells [22].

Another novel difluoro curcumin formulation was reported by Dandawate et al. (2012), generally called CDF and shown to have greater anticancer activity. The CDF conjugate with *β*-cyclodextrin (CDFCD) in 1:2 proportions exhibited significantly lower IC_50_ values when tested against a group of cancer cell lines—BXPC-3, MDA-MB-231, and PC-3 compared to CDF alone [23]. Further in vivo studies in mice revealed that the conjugate favourably accumulated in the pancreas, and the levels of CDF-*β*-cyclodextrin conjugate in the pancreas were 10 times higher than that in serum, following intravenous administration of an aqueous CDF-*β*-cyclodextrin preparation. These studies suggest that the self-assembly of *β*-cyclodextrin and CDF may significantly enhance the bioavailability and tissue distribution of these curcumin analogues [23]. Manju et al., in 2011, synthesised a polyvinyl pyrrolidone–curcumin conjugate to enhance the water solubility of curcumin [24]. Self-assembly of the drug conjugate was done in an aqueous solution to form nano-sized micellar aggregates, which were cationic and stable against hydrolytic degradation. The cytotoxic potential of the conjugate was evaluated against the L929 fibroblast cells, indicating that the conjugate has higher cytotoxicity than free curcumin, possibly due to its enhanced aqueous solubility and polymer-mediated drug internalisation [24].

Sohail et al. 2021 reported a study on the nanoformulation of dimethoxycurcumin (DiMC), also known as dimethylcurcumin, to improve its solubility and stability [25]. By complexation with hydroxypropyl-γ-cyclodextrin, the commercial curcumin containing DiMC could acquire increased solubility and stability. Recently, solid dispersions (SDs) of DiMC were prepared with polyethylene glycol (PEG) 4000, PEG 6000, and poloxamer 188 as carriers using the fusion method and polyvinylpyrrolidone (PVP K30) as a carrier using the solvent evaporation method, respectively. The formulation using PVP K30 at a ratio of 10:1 to DiMC was the best, where DiMC dispersed in an amorphous form with a cumulative dissolution of more than 83% in 5 min. These results showed that the drug dissolution rate could be improved significantly by utilising all SDs [25]. Another study by Wei et al. performed experiments to achieve dicarbonyl curcumin analogues via the aldol condensation reaction of aldehydes and ketones, achieving a final yield of above 50% for all novel compounds showing > 95% purity by HPLC analysis [26].

Additionally, numerous studies conducted by [10,16,25,27,28,29,30,31,32,33,34,35,36,37,38,39,40,41,42,43,44,45,46,47,48,49,50,51] reported a formulation of nanocurcumin in the form of a curcumin nanocrystal powder where the authors assessed its physiochemical properties, as well as the antibacterial, antioxidant, anticancer, and anti-inflammatory action. Nanocurcumin has reportedly been used as a novel drug delivery approach for biomaterials in dentistry. Azad and co-workers (2024) reported that methods utilising emulsifiers such as carbohydrate complexes, polyethoxylated hydrogenated castor oil, lipid complexes, phospholipid complexes, polysorbates, water-dispersible nanopreparations, and spray drying could also be used to increase the solubility of curcumin BioCurc, Cavacurcmin, CurcuWIN, Hydrocurc, Meriva, Nanocurcumin, Novasol, Theracurmin, and Turmipure Gold [27].

Even though a wide range of available literature suggests that these new strategies on curcumin-based nanoparticulate formulations demonstrate some promise of curcumin to be used as a therapeutic agent, the issue of the rapid metabolism of curcumin remains a matter of concern.

## 6. Structural Analogues of Curcumin and Their Anticancer, Antioxidant, and Anti-Inflammatory Activity

Curcumin is a symmetrical *β*-diketone and incorporates structural changes at different active sites (methylene group, diketo functionality, linker chain, and an aromatic/aryl side chain) [10]. Various researchers have conducted several studies to date to modify the structure of curcumin, simultaneously reforming its pharmacological efficacy. This review covers several structural modifications of curcumin targeting three active sites—an aromatic side chain, diketo functionality, and an active methylene group. Several analogues that could not be classified under the abovementioned groups have been separately studied for their in vivo and in vitro studies in the next section of the review. The following account elaborates on some of the literature reported in the past 15 years, focusing on the main reactive sites and the subsequent enhancement in their bioactivity. Potential anticancer, antioxidant, and anti-inflammatory studies indicating their IC_50_ values for each curcumin analogue are reported in Table 1.

**Scheme 1:**
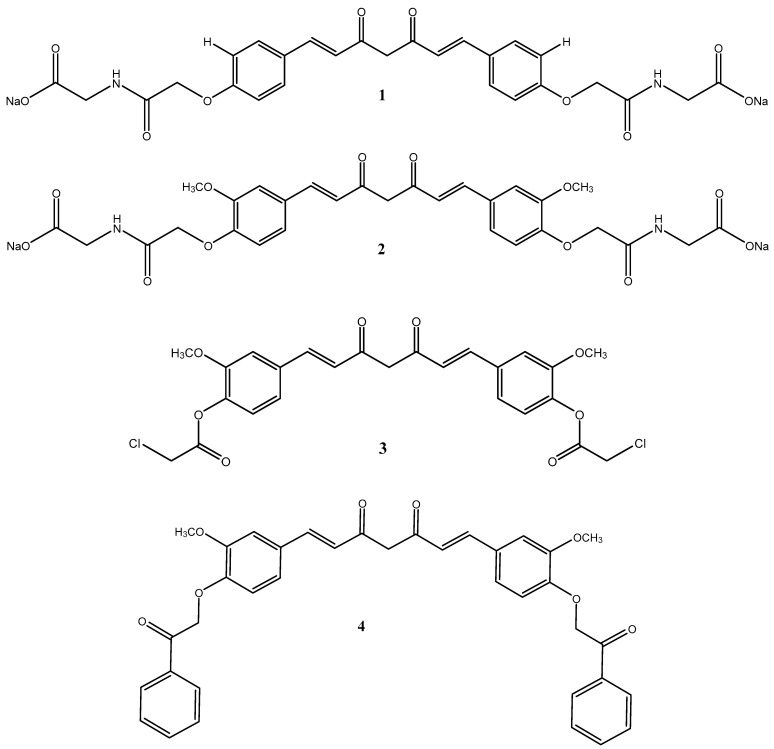

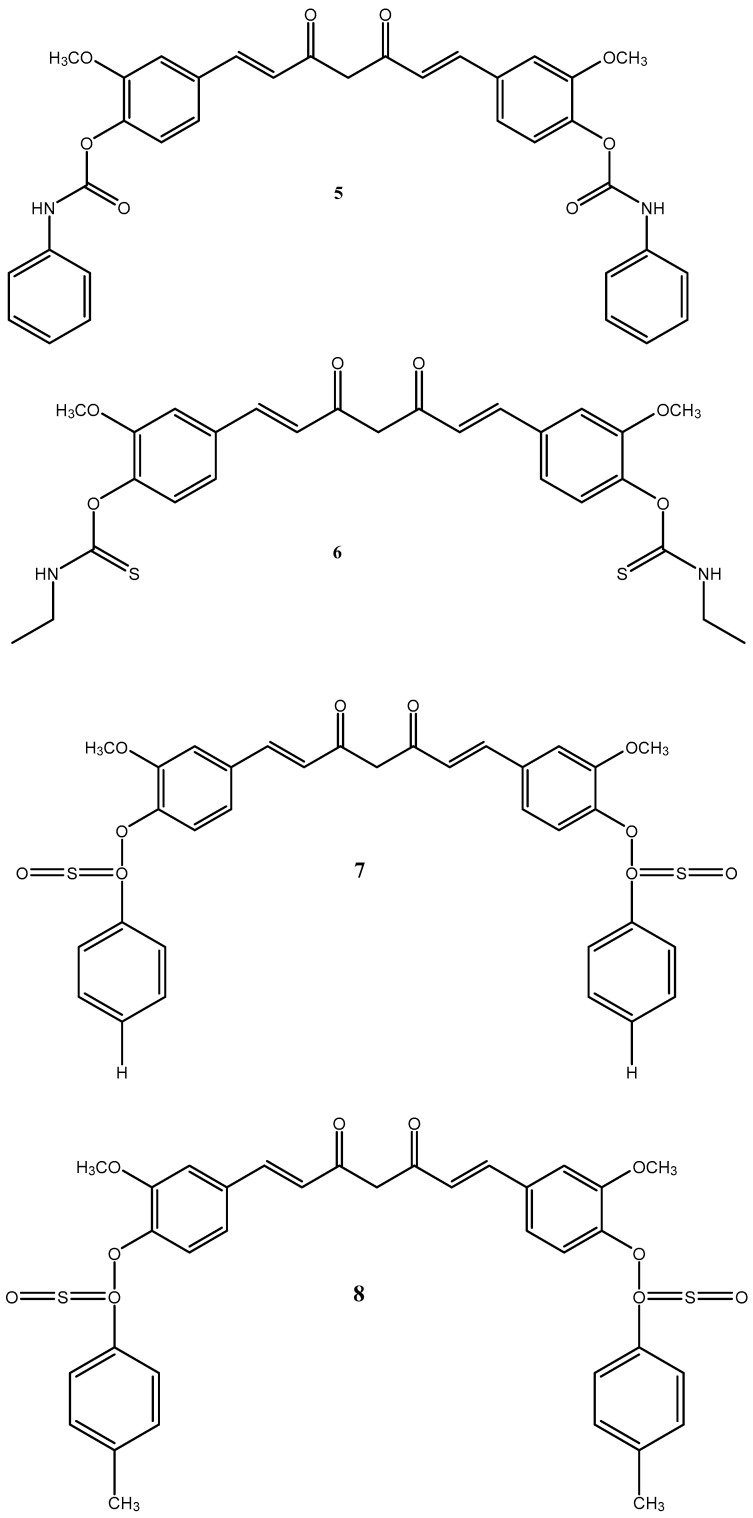
Structural analogues of curcumin (**1**–**8**).

Amide-containing curcumin analogues (**1**–**2**) were synthesised by Banuppriya et al. (2018), targeting aromatic side chains, which indicated enhanced water solubility [28]. The target molecule(s) was achieved by hydrolysis in the presence of sodium hydroxide and methanol (Scheme 1). Both compounds were tested for their anticancer activity, which indicated an inhibition in HeLa cancer cell growth, showing an increase in the p53 level. The compounds were compared to curcumin, which suggested enhanced aqueous solubility and in vitro stability. LogP analysis confirmed that the compounds possessed good lipophilicity, making them potential anticancer agents with a better tendency to cross the blood–brain barrier. El-Gazzar et al. (2016) reported six novel (**3**–**8**) (Scheme 1) symmetrical curcumin analogues through modifications to the aromatic side chain by substituting the phenolic -OH with different linkers [34]. The analogues reportedly demonstrated radioprotective potential by in vivo studies. A rats model was used to analyse these compounds, where whole-body gamma irradiation was performed on rats at a 7 Gy single dose. The blood samples were collected from the plasma to test for their antioxidant and oxidative stress markers. The rats also demonstrated decreased levels of proinflammatory markers such as IL-6, TNF-*α*, and NF-kB. The synthesised analogues by NF-kB inhibition confirmed the post-protective effect in this study.

**Scheme 2:**
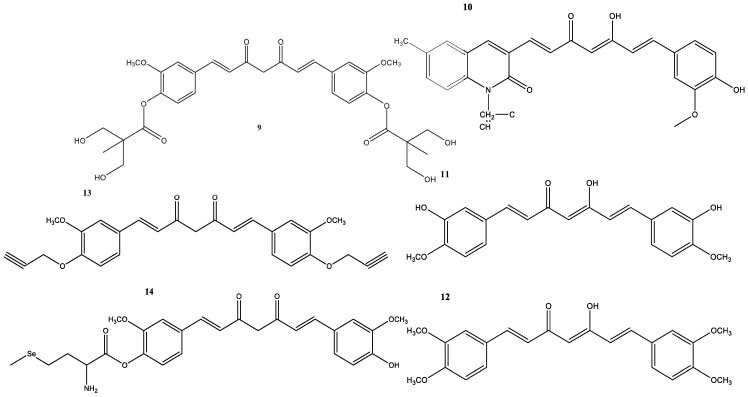
Structural analogues of curcumin (**9**–**14**).

Hsieh et al. (2017) synthesised various curcumin analogues where compound **9** indicated higher antiproliferative activity [39]. The compound was synthesised under an acid catalysis reaction using an appropriate ester at 45–50 °C in THF, forming a hydroxyl ester compound. Compound **9** indicated 10-fold potency when compared to curcumin both in vitro and in vivo, also indicating a synergistic activity when used with doxorubicin against a triple-negative breast cancer cell line [39] (Scheme 2). A series of novel curcumin quinoline hybrids from several substitutions of 3-formyl-2-quinolones and vanillin were synthesised by Raghavan et al. (2015) [44]. Compound **10** indicated the most potent activity when investigated for their cytotoxicity against a panel of representative cell lines: A549, MCF-7, SKOV3, and H460 (Scheme 2). Its potency was determined due to the morphological changes in the SKOV3 cell lines and apoptotic cell death by arresting the cells in the S and G2/M phases. In addition, six symmetric curcumin analogues by Ciochina et al. (2014) were synthesised by the condensation of the appropriate aldehydes with the acetyl acetone–boric oxide complex in ethyl acetate in the presence of tributyl borate and n-butylamine [32]. The analogues were investigated for their antioxidant and anticancer activity, where compounds **11** and **12** demonstrated a greater potential to be chemopreventive agents due to their low cytotoxic potential [32] (Scheme 2). Lopes-Rodrigues et al. (2017) synthesised compound **13** to understand the role of curcumin derivatives on P-glycoprotein (P-gp) indicators (Scheme 2). Synthesis was performed under reflux conditions using curcumin, and Cs_2_CO_3_ and Bu_4_NBr in the presence of acetone and propargyl bromide solution at 60 °C for 4 h, producing an orange solid product through recrystallisation as the final step [42]. The compound inhibited P-gp activity, caused cell cycle arrest at the G2/M phase, and increased cell death by apoptosis in a multidrug-resistant (MDR) chronic myeloid leukaemia cell line [42]. Furthermore, Cao et al., in 2014, synthesised three curcumin analogues through conjugation with bioactive compounds via ester bonds, where **14** (Scheme 2) showed the strongest antiproliferative activity when investigated in four cell lines, namely Hep G2, LX-2, SMMC7221, and MDA-MB-231, indicating IC_50_ values ranging from 0.18 to 4.25 μM [16].

**Scheme 3:**
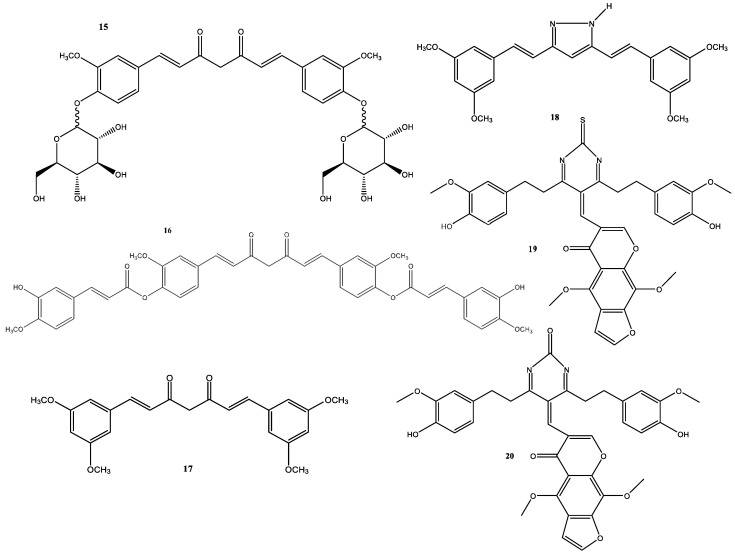
Structural analogues of curcumin (**15**–**20**).

Rao et al. (2014) synthesised and evaluated the biological activity of curcumin-b-di-glucoside and tetrahydrocurcumin-b-di-glucoside by a bi-phasic reaction medium [52]. The conjugated analogues showed a significant inhibition in colon and breast cancer cell lines against the MCF-7, HT-29, and A549 cell lines, where compound **15** indicated the best anticancer activity [52] (Scheme 3). In addition, several analogues were synthesised by Feng et al. (2015) by combining cinnamic acid and curcumin and their antioxidant, antibacterial, and anticancer activity evaluated. The analogue **16**, containing hydroxyl and methoxy groups as the active ingredient, indicated a greater anticancer activity compared to the other analogues [36] (Scheme 3). Kanwar et al. (2011) synthesised dimethoxycurcumin (**17**) and reported its anticancer properties against MCF-7 breast cancer cells, which confirmed cell death through cell cycle arrest and the induction of apoptosis [53] (Scheme 3). Lien and co-workers, in 2015, designed and synthesised several curcumin analogues and evaluated their ability to degrade the HER2 gene. Compound **18** was achieved via the modification of the diketo group of dimethoxycurcumin using acetic acid, indicating better ability than other curcumin analogues and curcumin in inhibiting the HER2 expression and further induction of G2/M cell cycle arrest followed by apoptosis [40] (Scheme 3). A series of novel heterocyclic curcumin analogues were designed by Borik et al. (2018), and their anticancer activity by MTT assay was evaluated amongst MCF-7 and HepG2, as well as the normal cell line HFB4 [31]. Out of the 14 novel analogues synthesised, 2 compounds (**19**, **20**) showed better anticancer activity than curcumin (Scheme 3). Synthesis was carried out by a one pot condensation reaction, an acid catalysed Biginelli reaction, with three components, i.e., furochromone carbaldehyde, curcumin, and urea, to give the corresponding derivatives. These analogues were compared against the already existing cancer drugs 5-fluorouracil and doxorubicin [31].

**Scheme 4:**
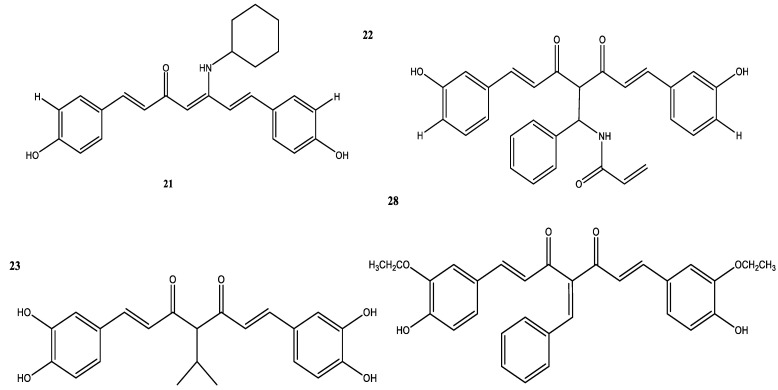

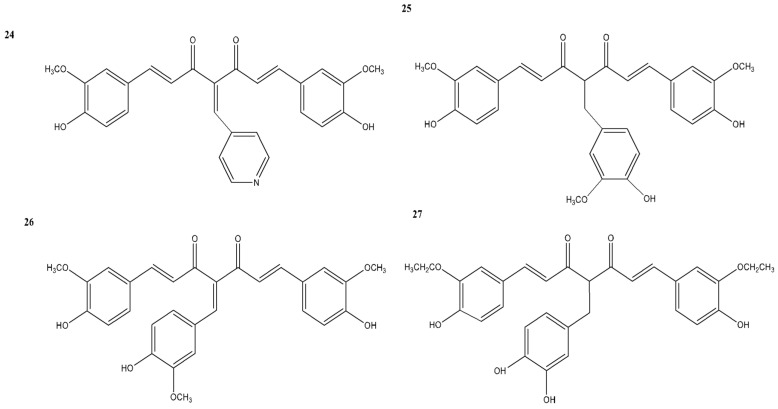
Structural analogues of curcumin (**21**–**28**).

Thirteen analogues of curcumin were synthesised by De Vreese et al. (2016), with a central *β* enaminone fragment substituting the *β*-diketone moiety to improve the solubility and bioactivity of curcumin utilising the microwave-assisted irradiation method. The cytotoxicity studies were performed amongst the EA.hy926, HT-29, and Caco-2 cell lines. Compound **21** was revealed to be the most potent amongst the other potent analogues, indicating strong cytotoxic effects [33] (Scheme 4). Hackler and co-workers in 2016 reported a remarkable study investigating both in vitro and in vivo anticancer effects of a curcumin analogue named C-150 (**22**) against glioma cells (Scheme 4). The analogue consisted of meta-hydroxyphenyl side chains and a *β*-phenyl- *β*-acryl-amido branched central motif. C-150 was studied for its cytotoxicity activity against eight glioma cell lines, GBM 1–6, U251 MG, and U373 MG to understand the role it plays in mediating transcription factors and proteins [38]. It was reported to be an effective NF-kB inhibitor in the in vitro model, and it proved to be 30 times more potent than curcumin in inducing the expression of genes and proteins in ER stress. The C-150 analogue was also studied using the in vivo model, which showed an increase in the survival of an experimental set of animal models compared to the vehicle control [38]. A dimethylated curcumin analogue (**23**) (Scheme 4) was identified and studied by Tu et al. (2017), which was substituted at the active methylene site, demonstrating to be the most potent amongst various curcumin analogues when tested for its cytoprotective activity against t-BHP-induced death of Hep G2 cells [48]. The compound also indicated increased stability, a mechanism dependent on activating the Nrf-2 signalling pathway [48]. Simultaneously, a curcumin analogue, 4-(4-Pyridinyl methylene) (**24**) (Scheme 4), having a modification at the active methylene site, was discovered by Fan and co-workers (2018) and was studied for its antitumor action. Due to its regulatory effects for Heat shock protein—Hsp90, it was identified as an active molecule for treating myeloid leukaemia [35]. Similarly, another curcumin analogue, 4-(4-hydroxy-3-methoxy-phenyl-methyl (C086) (**25**) (Scheme 4), having an active methylene site modification, was discovered by Wu et al. (2015). It also indicated that C086 was a Hsp90 inhibitor, revealing it could lead to the dual suppression of Abl kinase activity and Hsp90 chaperone function [49]. Rišiaňová and co-workers (2017) presented the synthesis and characterisation of three Knoevenagel condensates, and the analogues (**26**–**28**) (Scheme 4) were further studied for their cytotoxic effect in vitro, SOD mimetic, and GSH oxidation capacity in the human colon carcinoma cell line DLD-1. All the compounds exhibited an antiproliferative effect and a significant reduction in multidrug resistance, also indicating a decrease in SOD enzymes that exposes the tumour cells to more oxidative stresses [46].

**Scheme 5:**
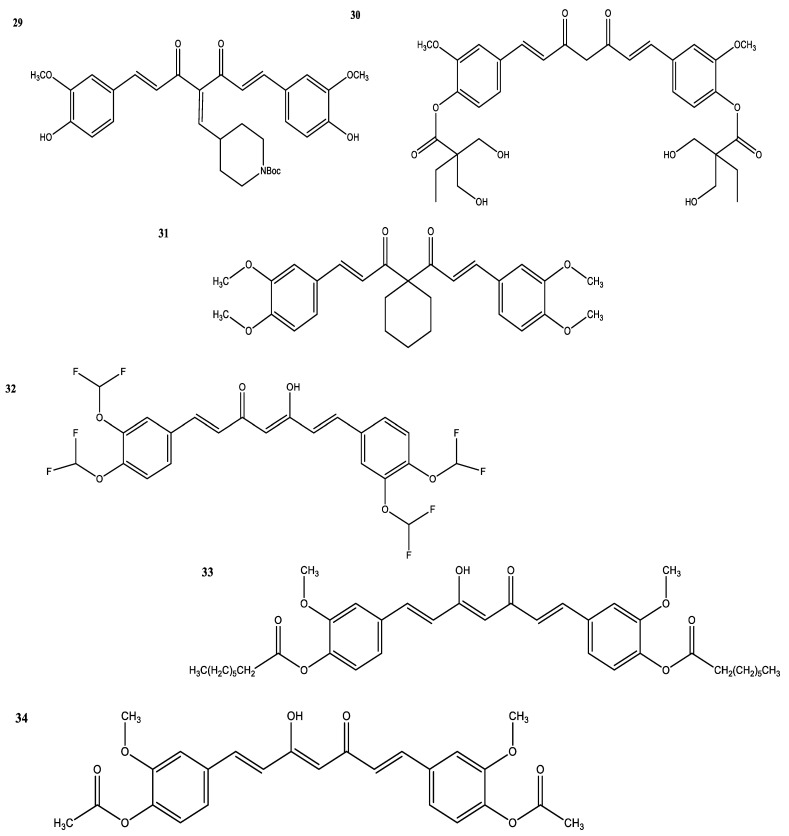
Structural analogues of curcumin (**29**–**34**).

Additionally, Srivastava et al. (2016) also investigated four Knoevenagel condensates of curcumin via a reaction with an aldehyde in the presence of DMF and a catalytic amount of piperidine. The analogues were tested for their antiproliferative activity in MCF-7 cell lines and their capacity to disrupt microtubules and induce p53-dependent apoptosis, where compound **29** (Scheme 5) demonstrated the highest apoptotic activity [47]. In 2023, Du et al. reported a synthesis of compound **30** (Scheme 5) by the esterification of curcumin in water and alcohol to increase its aqueous solubility. Compound **30** showed higher cytotoxicity, exhibiting 10 times higher potency against the doxorubicin-resistant MDA-MB-231 cell lines than curcumin. It also demonstrated a synergistic activity when used with doxorubicin against breast cancer. The compound further suppressed MDA-MB-231 TNBC cell invasion by regulating the MAPK/ERK/AKT signalling pathway and cell cycle [54]. Lu et al. (2023) recently reported a study on the synthesis of the diketone analogue of curcumin, FLLL32 (**31**) (Scheme 5), and (E)-3-(3,4-dimethoxyphenyl-1-[1-[(E)-3-(3,4-dimethoxyphenyl)prop2-enoyl] cyclohexyl]prop-2-en-1-one. FLLL32 targets the transcription factor STAT3 for tumour cell proliferation, survival metastasis, and drug resistance. It is known to be more selective in targeting than curcumin due to the two hydrogen atom replacements on the central carbon of curcumin with a spirocyclohexyl ring [43]. FLLL32 has been studied for its antiproliferation properties amongst human osteosarcoma 143.98.2 cells, showing a decrease in cell growth and a delay in tumour growth through the reduction of cell proliferation. The analogue also induced cell apoptosis in a mouse model of the 143.98.2 xenograft nude [43].

**Scheme 6:**
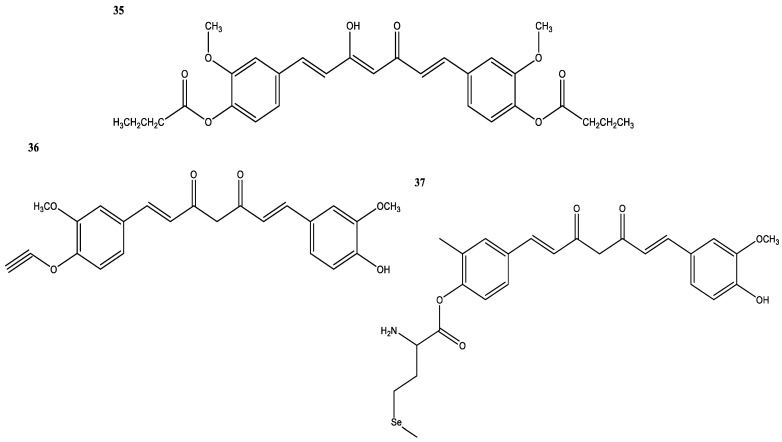

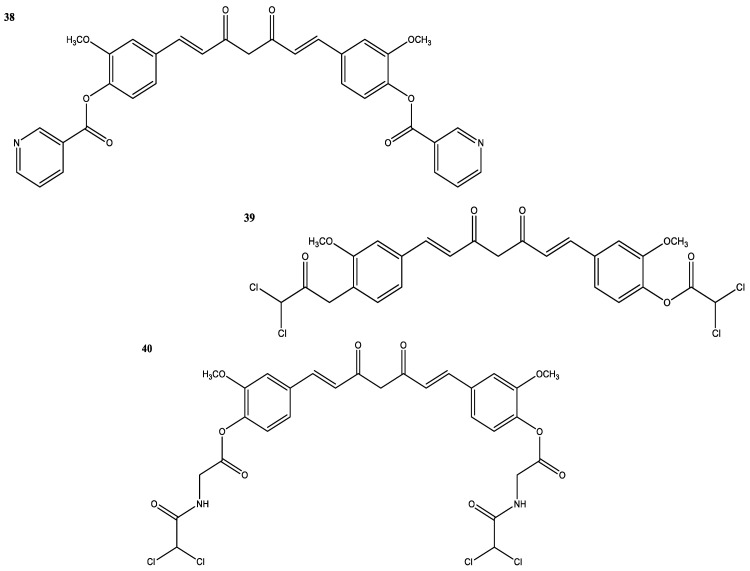
Structural analogues of curcumin (**35**–**40**).

Furthermore, Gong et al. (2024) reported curcumin analogues containing halogen atoms or nitrogen atoms containing substituents on the benzene rings. The analogues were prepared for castration-resistant prostate cancer cell inhibition, where compound **32** (Scheme 5) exhibited dose-dependent cytotoxicity against 22RV1 cells when analysed using the standard MTT ((3-(4,5-dimethylthiazol-2-yl)-2,5-diphenyltetrazolium bromide) method. The average half-maximum inhibitory concentration at 48 h and 72 h was reported to be 8.791 and 8.516 μM, respectively [37]. Furthermore, a study by Zarei et al. (2024) has recently reported the synthesis of curcumin dioctanoate (**33**) (Scheme 5), curcumin diacetate (**34**) (Scheme 5), and curcumin dibutanoate (**35**) (Scheme 6) by the reaction between curcumin and the corresponding aldehydrides in the presence of a catalytic amount of 4-dimethylamino pyridine (DMAP). Out of the three ester analogues of curcumin, curcumin dibutanoate (**35**) showed remarkably increased solubility in food grade oils. Compound **35** showed a potent antioxidant activity using the DPPH method and presented an antioxidant activity of 1.92%. Curcumin dibutanoate showed more stability against oxidation and heat when compared to curcumin [51].

Recently, a review highlighting the recent advances of curcumin analogues in breast cancer conducted by Yin and co-workers (2022) reported various studies on curcumin analogues with improved solubility and bioavailability compared to curcumin [50]. Compound **36** (Scheme 6) was synthesised through a substitution reaction involving the phenolic hydroxy group of curcumin. The analogue demonstrated growth in inhibition when tested for its cytotoxicity against the SUM149 and MDA-MB-231 cancer cell lines with IC_50_ values of 11.20 and 18.00 μM, respectively. It also showed significant potency in inducing cancer cell apoptosis and downregulating the NF-kB signalling pathway. Compound **37** (Scheme 6) was achieved by the synthesis of curcumin with selenomethionine, demonstrating a potent antiproliferative activity and improved bioavailability in MDA-MB-231 cells with an IC_50_ of 0.52 μM [50]. Compounds **38**–**40** (Scheme 6) were also synthesised and reported for their corresponding activity, as mentioned in the review by Yin et al. (2022) [50]. In 2019, compound **38** was synthesised and reported to have enhanced solubility and stability by combining curcumin and niacin. It exhibited antiproliferative activity in breast cancer cells and selectively induced G2/M cell cycle arrest and tumour cell apoptosis. Similarly, in 2022, compounds **39** and **40** were synthesised through the conjugation of curcumin and dichloroacetate, displaying high selectivity and significant activity on antiproliferative and anti-migrating activities against MDA-MB-231 cells with an EC_50_ value of 0.42 and 0.78 μM, respectively. Compound **40** also achieved a significant balanced property of solubility in water [50].

**Scheme 7:**
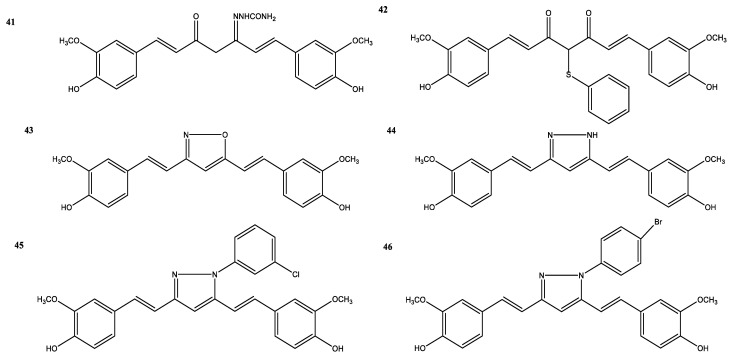
Structural analogues of curcumin (**41**–**46**).

Additionally, Yin et al. (2022) reported compounds (**41**–**46**) (Scheme 7) with modifications to their diketone and active methylene sites. Compound **41** modification was performed by replacing one of the *β*-carbonyl with a semi-carbazone moiety, demonstrating efficient antiproliferative activity against breast cancer MCF-7 cells in vitro. Similarly, compound **42** was synthesised by an original procedure via sulfenic acid condensation, which exhibited higher antiproliferation potency than curcumin against breast cancer MDA-MB-231 cells (IC_50_ = 15.00 μM). Compounds **43** and **44** were identified as isoxazole curcumin analogues and substituted at the diketone site. Compound **43** displayed more potent antitumor activity than curcumin in MCF-7 and MCF-7R cells, with IC_50_ = 13.10 and 12.00 μM, respectively. Compound **44** demonstrated high solubility and stability, which showed a significant effect in inhibiting STAT3 and was also found to be more potent than curcumin against MDA-MB-231 and MCF-7 cells, with IC_50_ = 3.37 and 2.56 μM, respectively. It was found to be in favour of suppressing cell migration and invasion and inducing apoptosis. In addition, compound **45**, a curcumin pyrazole analogue, was synthesised by the base-catalysed cyclisation of curcumin with the corresponding phenyl-hydrazine. It also induced cell death by arresting the cell cycle in the SubG1 phase and inducing cell damage by impairing the mitochondrial membrane potential against breast cancer MCF-7 cells (IC_50_ = 34.99 μM). Lastly, compound **46** was synthesised through the condensation of tetrahydrocurcumin with hydrazine, which was substituted with the 4-bromo-phenyl group at the pyrazole ring. It demonstrated antiproliferative activity against three representative cell lines using in vitro MTT assays. Compound **46** showed significant growth inhibition against MCF-7 cancer cell lines (IC_50_ = 5.80 μM) [50].

## 7. In Vitro and In Vivo Studies on the Antioxidant, Anti-Inflammatory, and Anticancer Activities of Curcumin and Its Analogues

Numerous in vitro investigations have consistently demonstrated the potent antioxidant properties of curcumin [55,56,57,58]. It can mitigate oxidative stress by scavenging free radicals and enhancing endogenous antioxidant defences [58]. Furthermore, in vitro studies have elucidated the anti-inflammatory effects of curcumin through the modulation of key inflammatory pathways and attenuation of proinflammatory mediator production. These findings have been further corroborated by in vivo studies. Various preclinical models of inflammation-associated diseases, including but not limited to arthritis, colitis, and neuroinflammatory disorders, highlight the therapeutic potential of curcumin and its analogues.

Moreover, both in vitro and in vivo studies support the anticancer activity of curcumin. They involve the suppression of tumour growth, apoptosis induction, inhibition of angiogenesis, and modulation of various signalling pathways implicated in carcinogenesis [3,4,59,60]. Collectively, these studies underscore the multifaceted pharmacological effects of curcumin and its analogues. This advocates for their exploration as promising candidates for developing novel therapeutics targeting oxidative stress, inflammation, and cancer.

### 7.1. Antioxidant Activity

The antioxidant potential of curcumin and its analogues has been extensively investigated in both preclinical and clinical studies. For instance, Chen et al. (2015) demonstrated the superior stability and DPPH scavenging activity of liposomal curcumin compared to free curcumin [61]. Additionally, Priyadarsini et al. (2014) explored the antioxidant activity of various curcumin metal complexes, highlighting their ability to chelate metal ions and scavenge free radicals [6]. Further investigations by Shen et al. (2007) [62] and Gorgannezhad et al. (2016) [63] revealed that certain curcumin complexes, such as Cur-Cu (II) and Cur-Mn (II), exhibited enhanced antioxidant properties compared to free curcumin, possibly through mechanisms involving proton or electron donations.

Moreover, preclinical studies have elucidated the involvement of various signalling pathways in curcumin’s antioxidant activity. For instance, Hatcher et al. (2008) [64] demonstrated that curcumin increased the antioxidant defence mechanisms in rats, while Feng et al. (2017) [17] showed its efficacy in reducing prostatic adenocarcinoma growth. Dall’Acqua et al. (2015) [65] found the effect of *Curcuma longa* L. extract (150 mg/kg of total curcuminoids) on healthy rats’ in vivo antioxidant effects. The experiment was carried out over 33 days, and changes in the metabolome of the 24-h urine samples were evaluated using 1H NMR and HPLC–MS. The results indicate that the oral administration of Curcuma extract to healthy rats has an in vivo antioxidant effect, as it decreases the urinary levels of allantoin, m-tyrosine, 8-hydroxy-2′-deoxyguanosine, and nitrotyrosine.

Another study by Jakubczyk et al. (2020) evaluated the effect of curcumin on oxidative stress markers. Four studies with a total of 308 participants were included in the meta-analysis [66]. The average curcumin dose was 645 mg/24 h, and the participants were supplemented with curcumin for an average of 67 days. Using curcumin significantly increased the total antioxidant capacity (TAC) and showed a tendency to decrease the concentration of malondialdehyde (MDA) in plasma. The study concludes that curcumin can reduce the MDA concentration and increase the total antioxidant capacity, indicating that it may help reduce oxidative stress [66]. Moreover, Salehi et al. (2021) assessed the effects of curcumin supplementation on inflammatory, oxidative stress markers, muscle damage, and anthropometric indices in women with moderate physical activity. The double-blind, placebo-controlled clinical trial was conducted on 80 women. The results indicated that 8-week curcumin administration could significantly improve serum C-reactive protein (CRP), total antioxidant capacity (TAC), malondialdehyde (MDA), lactate dehydrogenase (LDH) levels, body composition, and maximum oxygen uptake (VO2 max) [67].

Deshmukh et al. (2019) synthesised a library of 18 compounds from curcumin of *α*, *α*′-bis(1H-1,2,3-triazol-5-ylmethylene) ketones and evaluated them for their in vitro antitubercular and antioxidant activities against their respective strains [68]. The results showed that some of the compounds from the series displayed good antitubercular and antioxidant activities. Compound **8l**, as Deshmukh et al. reported in their study, was found to be the most active antitubercular agent, with a MIC value of 3.125 µg/mL against Mtb H37Rv. Compounds **8e** and **8m**, as referenced in a study by Deshmukh et al., also displayed potent antioxidant activities, with IC_50_ values of 15.60 and 15.49 µg/mL, respectively [68].

### 7.2. Anti-Inflammatory Activity

Inflammation is implicated in the pathogenesis of numerous diseases, and curcumin and its analogues have shown promising anti-inflammatory effects both in vitro and in vivo. Studies have indicated that curcumin inhibits various inflammatory pathways, including lipo-oxygenase and cyclo-oxygenase activities, nitric oxide production, and ROS generation in different cell types [64]. Kato et al. (2023) investigated the anti-inflammatory activities of curcumin solid dispersions (C-SDs) in rats. They observed that smaller particle sizes of C-SDs were associated with increased bioavailability and anti-inflammatory effects [69]. Khan et al. (2012) reported the potent antiarthritic activity of synthesised curcuminoids in rat models of acute carrageenan-induced paw oedema and chronic adjuvant arthritis with minimal toxicity [70].

Several clinical trials have also evaluated curcumin’s anti-inflammatory effects in patients with rheumatoid arthritis (RA) and metabolic diseases. Kato et al. also demonstrated improved disease activity scores in RA patients receiving curcumin alongside conventional treatment [69]. Similarly, Panahi et al. (2012) observed reductions in the serum cytokine levels after curcumin administration in patients with metabolic diseases [71].

Another recent review by Peng et al. (2021) delved into the complex physiological and pathological mechanisms contributing to inflammatory diseases, such as inflammatory bowel disease, psoriasis, atherosclerosis, and COVID-19 [72]. The study explored the anti-inflammatory properties of curcumin, its regulatory impact on these illnesses, and the latest findings on its pharmacokinetics (Table 2) [72]. Additionally, the review summarised further clinical trials investigating the anti-inflammatory effects of curcumin in disease treatment (Table 2) [72]. Patwardhan et al. (2011) investigated the anti-inflammatory activity of the synthetic curcumin analogue dimethoxy curcumin (DiMC) in murine and human lymphocytes. DiMC and curcumin suppressed the proliferation of murine splenic lymphocytes and the secretion of cytokines [73]. They also scavenged basal ROS and depleted GSH levels in lymphocytes. Thiol-containing antioxidants were found to play a role in their anti-inflammatory activity. Moreover, both DiMC and curcumin inhibited lymphocytes post-Con A activation of NF-κB and MAPK, and phytohemagglutinin induced proliferation and cytokine secretion by human peripheral blood mononuclear cells. The study demonstrated the potent anti-inflammatory activity of DiMC, which could be an alternative to curcumin due to its superior bioavailability and comparable efficacy [73]. In a recent review, Chainoglou and Hadjipavlou-Litina (2019) examined clinical trials utilising curcumin analogues and derivatives with anti-inflammatory activity [74]. Their analysis included publications from 2008 to 2018, which detailed the structural characteristics, functional groups, modelling studies, structure–activity relationship, and in vitro and in vivo biological evaluation of these agents. The results of their study are presented in Table 3 [74].

### 7.3. Anticancer Activity

Curcumin has garnered significant attention for its potential anticancer properties, alone and in combination with conventional chemotherapy agents. Recent studies have highlighted its ability to inhibit various stages of carcinogenesis, including angiogenesis, tumour promotion, and tumour growth [75]. Additionally, curcumin has synergistic effects when combined with certain anticancer drugs, offering a promising approach to cancer treatment [75,76].

Clinical trials have investigated the efficacy of curcumin in various cancer types, including skin lesions, oral submucosal fibrosis, myeloma, and colorectal cancer. After curcumin administration, Salehi et al. (2019) documented symptomatic relief and histological improvements in patients with skin lesions and oral submucosal fibrosis. Furthermore, curcumin demonstrated tumour growth inhibition and downregulation of inflammatory markers in patients with different cancers, although its efficacy in advanced pancreatic cancer remains limited. Moreover, a study by Luo et al. (2021) investigated the antiproliferative effects of four curcumin analogues on human gliomas. The four analogues, including curcumin (IC_50_ = 4.19 μM), bisdemethoxycurcumin (IC_50_ = 29.15 μM), demethoxycurcumin (IC_50_ = 30.03 μM), and dimethoxy curcumin (IC_50_ = 29.55 μM), were found to promote the sub-G1 phase, G2/M arrest, apoptosis, and ROS production in human glioma cells, with dimethoxy curcumin showing the most promise. Dimethoxycurcumin suppressed cell viability, migration and colony formation; induced sub-G1, G2/M arrest, apoptosis and ROS production and increased LC3B-II expression to induce autophagy, as this natural process helps maintain cellular health by breaking down and recycling damaged or unnecessary components. Furthermore, Luo et al. (2021) conducted a study where a series of curcumin analogue (1E,4E)-1-aryl-5-(2-((quinazolin-4-yl)oxy)phenyl)-1,4-pentadien-3-one derivatives were synthesised and screened for antitumor activities against a human gastric cancer cell line (MGC-803), human prostate cancer cell line (PC3), and human breast cancer cell line (Bcap-37) [77]. One of the compounds, **5f**, as stated in the study, was found to significantly inhibit the growth of cancer cells and induce cell apoptosis in MGC-803 cells, and it is less toxic to NIH3T3 normal cells. The study suggests compound **5f** should be further investigated as a potential anticancer drug candidate [78].

New bis(hydroxymethyl) alkanoate curcuminoid derivatives were synthesised and tested by Hsieh et al. (2017) for their inhibitory activity against various breast cancer cell lines [39]. Among all the compounds, compound **9a** displayed more significant inhibitory activity against TNBC cells than curcumin. It also demonstrated a considerable inhibitory effect against doxorubicin-resistant MDA-MB-231 cells, with ten-fold higher potency than curcumin. In the MDA-MB-231 xenograft nude mice model, compound **9a** demonstrated ten times more potent activity than curcumin when used alone. Combined with doxorubicin, compound **9a** displayed a synergistic effect against MDA-MB-231 breast cancer cells, as confirmed by microarray analysis. The delayed tumour growth observed with compound **9a** was attributed to G2/M phase arrest, autophagy, and apoptosis, leading to cancer cell death [39].

Liu et al. (2022) brought attention to the clinical trials of curcumin outlined in Table 2 [41]. These trials revealed that, apart from its positive effects, curcumin could negatively impact the heart, liver, kidneys, blood, reproduction, and immune system. While curcumin has potential health benefits, effectively managing its adverse effects during clinical application remains a significant challenge [41].

In conclusion, curcumin’s antioxidant, anti-inflammatory, and anticancer activities and its analogues have been extensively studied in preclinical settings, offering promising therapeutic potential for various diseases. However, further clinical studies to confirm curcumin’s efficacy, toxicity, and bioavailability need to be explored.

**Table 2:** Clinical studies of curcumin and curcumin analogues that have been reported in the past five years [41,72,79,80,81,82,83].

Condition or Disease	Intervention/Treatment	Research Output	References
Rectal Cancer	Curcumin po bid twice daily with radiation therapy and capecitabine for 11.5 weeks to improve your health and fight your condition effectively.	To determine whether curcumin can enhance the sensitivity of tumour cells towards radiation therapy.	[41]
Colorectal Cancer Patients with Unresectable Metastasis	Curcumin 100 mg po bid (+Avastin/FOLFIRI)	To evaluate treatment efficacy, measure progression-free survival, overall survival rate, and overall response rate, and assess safety and level of fatigue.	[41]
Colon Cancer	500 mg of curcumin po bid orally twice daily for 2 weeks. Patients continue curcumin at the same dose for an additional 6 weeks during 3 cycles of 5-Fu treatment.	To evaluate the safety and effectiveness of a treatment and determine the response rate.	[41]
Advanced Breast Cancer	The treatment plan involves administering Paclitaxel in combination with either Curcumin or a placebo. The dosage of Paclitaxel is 300 mg, Given intravenously once a week for a duration of 12 weeks.	To evaluate the negative effects, overall well-being, duration without disease progression, and duration until treatment failure.	[41]
Metabolic Syndrome	Nanomicelle curcumin or placebo	To investigate the impact of nano micellar curcumin on blood glucose levels, lipid profile, blood pressure, and anthropometric measurements.	[41]
Prostate Cancer	Curcumin or placebo (500 mg po bid)	To evaluate the effectiveness.	[41]
Invasive Breast Cancer	Curcumin 500 mg should be taken orally twice a day, starting from the day surgical resection is scheduled, up to the night before surgery.	To investigate whether curcumin induces any biological alterations in the primary tumours of patients suffering from breast cancer.	[41]
Cervical Cancer	Cisplatin in combination with teletherapy plus high or low-rate brachytherapy and Curcugreen (BCM95) or placebo 2000 mg daily (each 6 h).	To evaluate the effectiveness and safety.	[41]
Metabolic syndrome	Curcumin 1 g daily 8 weeks	↓ TNF-*α*, IL-6, TGF-*β* and MCP-1	[72]
Male factor infertility	Curcumin nano micelle 80 mg daily 10 weeks	↓ CRP, TNF-*α*	[72]
Crohn’s disease	Theracurmin^®^ 360 mg daily 12 weeks	The treatment has shown significant clinical and endoscopic effectiveness while maintaining a favourable safety profile.	[72]
Irritable Bowel Syndrome	IQP-CL-101 contains 330 mg curcuminoids & essential oils per soft gel. Take 2 daily for 8 weeks.	Patients suffering from abdominal pain and discomfort may benefit from this treatment, as it can improve IBS symptoms and quality of life.	[72]
Osteoarthritis	Sinacurcumin^®^ 80 mg daily 3 mouths	↓ Visual Analogue Score (VAS), CRP, CD4+ and CD8+ T cells, Th17 cells and B cells frequency	[72]
Knee osteoarthritis	500 mg of *Curcuma longa* extract (CL) twice a day along with Diclofenac for 4 months.	It suppresses inflammation and brings clinical improvement in patients with KOA, which may be observed by decreased levels of IL-1*β* and VAS/WOMAC scores, respectively.	[72]
Knee osteoarthritis and knee effusion-synovitis	*Curcuma longa* extract 2 capsules of CL daily 12 weeks	CL was more effective than placebo for knee pain but did not affect knee effusion–synovitis or cartilage composition.	[72]
Knee osteoarthritis	Herbal formulation “turmeric extract, black pepper, and ginger” Curcumin (300 mg), twice a day 4 weeks	↓ PGE2	[72]
Knee osteoarthritis	Theracurmin^®^ Six capsules of Theracurmin per day 6 months	The treatment has shown promising potential for effectively treating knee osteoarthritis in humans.	[72]
Knee osteoarthritis	400 mg of a Curcumagalactomannoside complex called CurQfen daily for 6 weeks.	The treatment exerted beneficial effects in alleviating the pain and associated symptoms.	[72]
Osteoarthritis	CuraMed^®^ Curamin^®^ capsules containing 500 mg of curcuminoids (333 mg curcuminoids per capsule) or 500 mg of curcuminoids and 150 mg boswellic acid (per capsule) should be taken orally, three times a day for 12 weeks.	Reduces pain-related symptoms in patients with OA.	[72]
Knee osteoarthritis	LI73014F2 200, 400 mg/day 90 days	OA patients have reported significant pain relief, improved physical function, and an overall improvement in their quality of life.	[72]
Rheumatoid arthritis	Curcumin orally, 500 mg twice daily for 8 weeks.	There has been an improvement in both the DAS and ACR scores, indicating an overall improvement.	[72]
Psoriasis	Curcuminoid C3 Complex—4.5 g for 12 weeks.	The low response rate may have been due to a placebo effect or the natural history of psoriasis.	[72]
Major Depression	Curcumin intake should be 500–1500 mg per day for a period of 12 weeks.	Significant antidepressant effects.	[72]
Non-alcoholic fatty liver disease (NAFLD)	active ingredients formulated as soft gel capsules. 2 capsules/day 3 months	Increased cholesterol, increased glucose, decreased Aspartate transaminase (AST)	[72]
COVID-19	ArtemiC oral spray day 1 and day 2 twice daily	Increased clinical improvement, SpO2 normalisation, decreased O_2_ supplementation, decreased fever, decreased hospital stays.	[72]
COVID-19	SinaCurcumin, a supplement containing 40 mg of curcumin, should be taken twice daily for a period of two weeks.	Significantly improve recovery time.	[72]
COVID-19	Two tablets of curcumin (525 mg) with piperine (2.5 mg) daily for 14 days.	Substantially reduce morbidity and mortality while easing logistical and supply-related burdens on the healthcare system.	[72]
Dry eye syndrome	LCD capsule 1 tablet/day 8 weeks	There was an increase in Schirmer’s strip wetness length, tear volume, TBUT score, and SPEED score. Additionally, there was a decrease in OSDI score, corneal and conjunctival staining score, tear osmolarity, and MMP-9 positive score.	[79]
Healthy subjects	iron + HydroCurc 18 mg +500 mg/day 65 mg +500 mg/ Day 6 weeks	Decreased levels of TBARS, TNF-*α*, GI side effects, fatigue, and IL-6 were observed.	[83]

**Table 3:** Curcumin analogues and their in vitro and in vivo antioxidant, anticancer, and anti-inflammatory activity [72,74,84,85,86].

Structure or Functional Groups	Molecular Pathway Affected-Action Mechanism	In Vitro Assay (Cell Lines) and In Vivo Assay (Animal Models) (Ex Vivo Assay)	References
Removal of phenyl ring at the 7th position of the heptadiene backbone and addition of hydroxyl group CBA-iR: bis-demethylcurcumin (BDC)	NF-KB	Human myeloid leukemic cell line: KBM-5 Human prostate cancer cell line: PC-3. Human multiple myeloma cell line: U266. Human colorectal cancer cell line: HCT-116. Human breast cancer cell line: MCF-7.	[74]
Heterocyclic curcumin analog CBA-iR: BAT3	NF-KB	Murine fibrosarcoma cells: L929A	[74]
EF-31, EF-24	NF-KB	Mouse RAW 264.7 macrophage cells A human ovarian carcinoma cell line: A2780 A mouse mammary carcinoma cell line: EMT6	[74]
Symmetrical curcumin analogues CBA-iR: 2	NF-KB	Wistar rats	[74]
Phenolic 1,3-diketones have been identified as CBA-iR, which includes bis-dimethoxycurcumin (GG6) and its cyclized pyrazole analogue (GG9).	TLR4, NF-KB	Ba/F3 cells	[74]
Dimethoxycurcumin (DiMc) Compound that contains an increased number of methoxy groups and a conjugated double bond.	iNOS, NO, and NF-κB	Murine and human macrophage cell lines: RAW264.7	[74]
Curcuminoids dimethoxycurcumin (DMC), THC, DiMc and *bis-*dimethoxycurcumin (BDMC): -two methoxy groups and two hydroxy groups but lacks conjugated double bonds in the central seven-carbon chain -*α*, *β*-unsaturated carbonyl group	Heme Oxygenase-1	Murine and human macrophage cell lines: RAW264.7	[74]
Mono-carbonyl analogues of curcumin A03, A13, B18, CBA-iR: A01, and C22	iNOS, p65, TNF-*α*, IL-1*β*, IL-6, MCP-1, COX-2, PGES, and NF-ΚB	Mouse J774A.1 macrophages	[74]
A13	NO, TNF-*α*, and IL-6,	Mice	[74]
GL63	COX-2	H460 cells	[74]
Dibenzoylmethane (DBM): (2,2′-diOAc-DBM)	COX-2	TPA-induced CD-1 mice ear oedema	[74]
Unsymmetrical monocarbonyl curcumin analogues with dimethoxy group, furanyl ring, and vanillin moiety are represented by CBA-iR	The signalling pathway responsible for the generation of Prostaglandin E2.	Murine and human macrophage cell lines RAW264.7 and U937	[74]
The dienone functional group was modified into a monoketone through pharmachophore modification. Additionally, the side chain of aromatic rings was altered by incorporating symmetrical or asymmetrical substituents.	COX-2	-	[74]
Derivatives of curcumin with unsymmetrical dicarbonyl CBA-iR have been synthesized, including **17f**.	COX-2, PGE2.	Murine macrophages cell line: RAW264.7	[74]
-Analogues of pyrazole and isoxazole, denoted as CBA-iR: 4, 7.	COX-2, COX-1	-	[74]
CBA-iR: HP109/HP102 -there is either a methoxy or a methyl ester group attached to the phenyl ring.	COX-1	Jurkat T-cells	[74]
A group of cyclic analogues and 1,5-diphenyl-1,4 pentadiene-3-ones with OH-groups situated in the para position of the phenyl rings and various meta substituents.	COX-2, COX-1	-	[74]
There is an amide ring with an electron withdrawing substituent, as well as a trifluoromethyl substituent. The CBA-iR includes **5f**, **5j**, **5m**, **5h**, **5b**, and **5d**.	TNF-*α*, COX-2, and IL-6	Human non cell lung carcinoma: Calu-1 Human colon carcinoma: HCT 116 Human renal cell carcinoma: ACHN Human pancreas carcinoma: Panc1 Human non cell lung carcinoma: H460	[74]
*α*,*β*-unsaturated carbonyl-based compounds -N-methyl-4-piperidone and 4-piperidone moieties CBA-iR: **3**, **4**, **12**, **13**, **14**	sPLA2, COX-1, LOX, IL-6, and TNF-*α*	Murine macrophages cell line: RAW264.7	[74]
A group of new curcumin diarylpentanoid analogues have been developed. -N-methyl-N-(2-hydroxyethyl)-4-amino -diethyamine group at position 4 of the phenyl ring -2-methyl-N-ethyl-N-(2-cyanoethyl)-4-amino	COX, LOX, PLA2, and mPGES-1.	-	[74]
Mono-carbonyl curcumin analogues with an acryloyl group. -1-naphthalene CBA-iR: **1b** (BAT1)	ALR2, LOX	Breast Cancer: MCF7 and Central Nervous System, glioma: SF268 Non-small cell lung cancer: NCI-H460 Fisher-344 rats	[74]
C66	COX-2, IL-1*β*, TNF-*α*, IL-6, IL-12, and iNOS	The research involved studying Mouse primary peritoneal macrophage (MPM) cells, as well as C57BL/6 mice and Sprague–Dawley (SD) rats.	[74]
C66	-	Mice	[74]
Curcumin-related diarylpentanoid analogues -2,5-dimethoxylated and 2 hydroxylated phenyl groups CBA-iR: **2**, **13**, **33**	NO	Murine macrophages cell line: RAW264.7	[74]
Analogues of Diarylpentanoid: CBA-iR: **88**, **97**	NO	Murine macrophages cell line: RAW264.7	[74]
Analogues of Diarylpentanoid CBA-iR: 5-methylthiophenyl-bearing analogue	NO	Murine macrophages cell line: RAW264.7	[74]
Derivatives of Pentadienone oxime ester CBA-iR: **5j**	COX-2, iNOS, and NO, IL-6	Murine macrophages cell line: RAW264.7	[74]
N-substituted 3,5-bis(2-(trifluoromethyl)benzylidene)piperidin-4-ones CBA-iR: c6 and c10	IL-1*β*, TNF-*α*, IL-6, PGE2, NO	-Murine macrophages cell line: RAW264.7 -Rat	[74]
Analogues of Curcumin that contain one carbonyl group: -electron withdrawing groups in the benzene ring CBA-iR: AN1 and B82	IL-6 and TNF-*α*	Murine macrophages cell line: RAW264.7	[74]
Analogues of curcumin that contain a mono-carbonyl and a 5-carbon linker have been developed. These analogues possess a N, N-dimethyl pro-poxy substituent. CBA-iR: B75 and C12	IL-6 and TNF-*α*	Murine macrophages cell line: RAW264.7	[74]
EF24	TNF-*α* and IL-6	JAWS II dendritic cells (DCs)	[74]
EF24	-	Sprague–Dawley rats	[74]
EF24	TNF-*α* and IL-6	LPS-stimulated dendritic cells -rat	[74]
EF24	NF-ΚB, IL-1R	JAWS II dendritic cells (DCs)	[74]
EF24	NF-ΚB, COX2	-Rat	[74]
EF24	NF-ΚB, COX-2	B cells -Rat	[74]
A series of curcumin analogues CBA-iR: **5c**, **5b**, **5j**, **5g**, **5h**	trypsin, b-glucuronidase, TNF-*α*, IL-6	CCK-8 cells	[74]
C-5 Curcumin analogues	TNF-*α*/NF-ΚB pathway	Chronic myeloid leukemia cell line: KBM5 Colon cancer cell line: HCT116	[74]
R1 has NO2 present while R2 has either a methoxy/hydroxy group present and both are present on the cyclohexanone molecule. CBA-iR: C26	IL-6 and TNF-*α*	Mouse primary peritoneal macrophages (MPM cells) -ICR mice and Sprague–Dawley (SD) rats	[74]
Resveratrol-curcumin hybrids CBA-iR: a18	IL-6 and TNF-*α*	Murine macrophages cell line: RAW264.7 -C57BL/6 mice	[74]
Diarylpentadienone derivatives CBA-iR: **3i**	IL-6 and TNF-*α*	Murine macrophages cell line: RAW264.7	[74]
*β*-ionone-derived curcumin analogues CBA-iR: **1e**	IL-6 and TNF-*α*	Murine macrophages cell line: RAW264.7 -C57BL/6 mice	[74]
Cyclohexanone and 3′-methoxy CBA-iR: **3c**	IL-6 and TNF-*α*	Mouse J774.1 macrophages	[74]
Analogues of curcumin that contain only one carbonyl group: 2,6-dimethyl-2-propoxy and alkoxyl substituent.	IL-6 and TNF-*α*	Macrophages	[74]
Asymmetrical monocarbonyl analogues of curcumin CBA-iR: **3a**, **3c**	IL-6 and TNF-*α*	Murine macrophages cell line: RAW264.7 -C57BL/6 mice	[74]
Asymmetric mono-carbonyl analogues of curcumin (AMACs) CBA-iR: **3f**	IL-6 and TNF-*α*	Mouse primary peritoneal macrophages (MPM cells) -C57BL/6 mice	[74]
PAC	IL-10 and IL-4	Primary breast cancer cell culture: BEC114 Breast cancer cells: MDA-MB231, MCF-10A, MCF-7 and T-47D -Balb-c mice	[74]
Benzylidenecyclopentanoneanaloguesofcurcumin CBA-iR: hydroxylmethoxybenzylidenecyclopentanone analogue of curcumin	Histamine	Rat basophilic leukemia cells: RBL-2H3	[74]
1,5-bis(4-hydroxy-3-methoxyphenyl)-1,4-pentadien-3-one (hylin)	MRP5	HEK293 cells	[74]
Diarylheptanoids	PGE2	3T3 cells	[74]
Enone analogues of Curcumin: -7-carbon dienone spacer, -5-carbon enone spacer with and without a ring-3-carbon enone spacer	Nrf2	Nrf2-ARE reporter-HepG2 stable cell line	[74]
FN1 (3E,5E)-3,5-bis(pyridin-2-methylene)-tetrahydrothiopyran-4-one	Nrf2	Human hepatocellular cell line: HepG2-C8 -TRAMP mice	[74]
1,3-dicarbonyl and acyclic series	TRP channels	HEK293 cells	[74]
Compounds containing tetrahydroxyl groups are as follows: A2, which is 2,6-bis(3,4-dihydroxybenzylidene)cyclohexanone; B2, which is 2,5-bis(3,4-dihydroxybenzylidene)cyclopentanone; C2, which is 1,5-bis(3,4-dihydroxyphenyl)-1,4-pentadiene-3-one; and D2, which is 3,5-bis(3,4-dihydroxybenzylidene)-4-piperidone.	ALR2	-	[74]
Curcumin analogues (MACs) CBA-iR: **17** and **28**	MD2	Human Umbilical Vein Endothelial Cells (HUVECs) Murine macrophages cell line: RAW264.7	[74]
Asymmetrical pyrazole curcumin analogues	-	-	[74]
Rosmarinic acid, tetrahydrocurcumin, dihydrocurucmin, and hexahydrocurcumin	Phospholipase A2	-	[74]
2,6-bis (3,4-dihydroxybenzylidene) cyclohexanone CBA-iR: A2	-	Murine macrophages cell line: RAW264.7 -Mice	[74]
Curcumin	↓ NO, IL-1*β*, IL-6, iNOS ↑ IL-4, IL-10, Arg-1 promoted microglial polarisation to the M2 phenotype	LPS-induced BV2 cells	[72]
Curcumin	↓ IL-1*β*, IL-6, iNOS, and TNF-*α*, CD86 protein, ↑ IL-10, TGF-*β* ↓ TLR4 signalling	Subarachnoid haemorrhage mice models	[72]
Curcumin	↑ PPAR-γ, ↓ NF-κB	Cigarette smoke extract-treated Beas-2B cells	[72]
Curcumin	↑ PPAR-γ, ↓ NF-κB, inflammation score ↓TNF-*α*, IL-6	Cigarette smoke-induced COPD rat models	[72]
Curcumin	↓ MCP-1, IL-17	Gp120-induced BV2 cells	[72]
Curcumin	↓ MCP-1, TNF-*α*, iNOS, NO ↓ ROS	LPS-induced inflammation in vascular smooth muscle cells	[72]
Curcumin	↓ TNF-*α*, IL-6 ↓ ROS	Palmitate-induced inflammation in skeletal muscle C2C12 cells	[72]
Curcumin	↓ TLR4, NF-κB, IL-27	TNBS-Induced Colitis Rats	[72]
Curcumin	↓ IL-6, IL-17, IL-23 ↑ IL-10 regulating the Re-equilibration of Treg/Th17	dextran sulphate sodium-induced colitis mice	[72]
Curcumin	↓ IL-1*β*, IL-6, MCP-1	DSS-induced colitis mouse model	[72]
Curcumin	↓ TNF-*α*, IL-6	DSS-induced ulcerative colitis mice model	[72]
Curcumin	↓ TNF-*α*, IL-6, IL-17 ↑ IL-10	DSS-induced acute colitis in mice	[72]
Curcumin Curcumin nanoparticles	↓ MMP-1, MMP-3, MMP-13, ADAMTS5, IL-1*β*, TNF-*α*	Post-traumatic osteoarthritis mouse model	[72]
Acid-activatable curcumin polymer	↓ IL-1*β*, TNF-*α*	Monoiodoacetic acid-induced osteoarthritis mouse model	[72]
Curcumin	↓ TNF-*α*, IL-17, IL-1*β* and TGF-*β*	Collagen-induced rat arthritis model	[72]
Curcumin	↓ IL-1*β*, TNF-*α*	Anterior cruciate ligament transection rat model	[72]
Curcumin loaded hyalurosomes	↑ IL-10 ↓ IL-6, IL-15, TNF-*α*	Fibroblast-like synovial cells	[72]
Curcumin	↓ IL-1*β*, TNF-*α*, NLRP3, caspase-1	Primary macrophages from the abdominal area of rats were used to induce gouty arthritis in a rat model using MSU.	[72]
Curcumin	↓ IL-17, TNF-*α*, IL-6, IFN-γ	Imiquimod-induced differentiated HaCaT cells	[72]
Curcumin	↓ IL-12, IL-22, IL-23, IFN-γ, TNF-*α*, IL-2	Transgenic mouse model of psoriasis	[72]
Curcumin	↓ IFN-γ	TPA-induced K14-VEGF transgenic psoriasis	[72]
Curcumin nanohydrogel	↓ TNF-*α*, iNOS	imiquimod-induced psoriasis model	[72]
Curcumin	↓ IL-1*β*, IL-6, TNF-*α*, NF-κB ↓stressed-induced P2X7R/NLRP3 inflammasome axis activation	Chronic unpredictable mild stress-induced rat model	[72]
Curcumin	↓ TNF-*α*, IL-6	Chronic unpredictable mild stress-induced rat model	[72]
Curcumin	↓ IL-1*β*	CUMS depression model	[72]
Curcumin	↓ TNF-*α*, VCAM-1, TLR4, IL-1*β*, NF-κB, ICAM-1	ApoE mice	[72]
Mannich Curcuminoids	↓ NF-κB, IL-6, IL-4, TNF-*α*	TNBS-induced colitis rats’ model	[72]
TRB-N0224	↓ TNF-*α*, MMP-9, MMP-13, IL-1*β*, IL-6	A model of osteoarthritis induced by anterior cruciate ligament transection in rabbits.	[72]
Curcumin analogue AI-44	↓TNF-*α*, IL-1*β*	MSU-induced THP-1 cell	[72]
Curcumin diglutaric acid	↓ TNF-*α*, iNOS, COX-2, NO, IL-6	LPS-stimulated RAW 264.7 macrophage cells	[72]
Curcumin-galactomannoside (CGM)	↓ COX-2, PGE2, iNOS, TLR4, IL-6, TNF-*α*	Acetic acid-induced colitis	[72]
Next Generation Ultrasol Curcumin (NGUC)	↓ COMP, NF-κB CRP, MMP-3, TNF-*α*, IL-1*β*, IL-6, 5-LOX, COX-2	MIA-induced OA	[72]
Curcumin	↓ IL-1*β*, IL-6, TNF-*α*, p53, CRP	Mice	[84]
Demethoxycurcumin	↓ TNF-*α*, NO, IL-4, IL-13, IL-1*β*, IL-6	N9 microglial cells	[85]
Curcumin-loaded tetrahedral framework nucleic acids (Cur-TFNAs)	↓ NO, IL-6, IL-1*β* and TNF-*α*, NF-κB, ROS	RAW264.7 cells	[86]

↓ Decrease ↑ Increase.

## 8. Future Directions

In this review, we have highlighted the development of new curcumin analogues to improve their solubility, stability, and bioavailability for medical applications. We have also focused on their antioxidant, anti-inflammatory, and anticancer activities.

Although many curcumin analogues have been studied over the years, poor bioavailability is still a major limitation to the therapeutic application of curcumin. The use of nanotechnology and a targeted drug delivery system, such as the protection of various active sites of curcumin by reacting it with known metal complexes for the modification of the chemical structure of curcumin, offers opportunities for developing new formulations to enhance curcumin’s solubility and bioavailability. Various drug delivery systems have already been shown to improve the cellular uptake, tissue specificity, and effectiveness of curcumin. Nevertheless, most curcumin analogues are still preclinical; further studies at the preclinical and clinical stages are needed to confirm their efficacy and safety in patients. On the other hand, further studies on the synergistic effects of curcumin and its analogues with the current therapeutic drugs may also help improve the efficacy and safety of these drugs for treating various diseases and conditions, including inflammatory diseases and cancer. Currently, our group is modifying the structure of curcumin, focusing on the aromatic group/aryl side chain and introducing various other therapeutic agents to the known active site. We aim to achieve numerous curcumin analogues from various reactions under different conditions. The rationale behind using these methods is to develop and produce new drug delivery systems, augmenting the solubility and bioavailability of curcumin.

## Figures and Tables

**Table 1:** Biological activities of curcumin analogues—anticancer, antioxidant, and anti-inflammatory.

Compound(s)	Biological Activity			Cell Line Tested (IC50 Value—μM)	References
	Antioxidant	Anticancer	Anti-Inflammatory		
**1**	–	+	–	HeLa—0.5 ± 0.003	[1]
**2**	–	+	–	HeLa—0.5 ± 0.005	[1]
**3**	+	–	+	Not reported	[2]
**4**	+	–	+	Not reported	[2]
**5**	+	–	+	Not reported	[2]
**6**	+	–	+	Not reported	[2]
**7**	+	–	+	Not reported	[2]
**8**	+	–	+	Not reported	[2]
**9**	–	+	+	MDA-MB-231—2.67 ± 0.18 HCT-116—3.91 ± 0.27 PC-3—3.90 ± 0.08	[3]
**10**	–	+	–	A549—23.9 ± 2.5 MCF-7—36.2 ± 1.99 SKOV3—12.8 ± 0.21 H460—21.75 ± 0.55	[4]
**11**	+	+	–	SKOV3—5.58 ± 2.0	[5]
**12**	+	+	–	SKOV3—3.51 ± 0.74	[5]
**13**	+	+	–	H460—3.4 ± 0.84 RH460—2.6 ± 0.25 K562—6.3 ± 0.95 K562 Doxorubicin—2.73 ± 0.61	[6,7]
**14**	–	+	–	Hep G2—0.31 LX-2—0.62 SMMC-7721—0.81 MDA-MB-231—0.52	[8]
**15**	–	+	–	HT29—41.56	[9]
**16**	–	+	–	MCF-7—0.51 Hep G2—0.58 LX-2—0.63 3T3—0.79	[10]
**17**	–	+	–	MCF-7	[11]
**18**	–	+	–	Not reported	[12]
**19**	–	+	–	Hep G2—23	[13]
**20**	–	+	–	MCF-7	[13]
**21**	–	+	–	Caco-2—7.8 HT-29—4 EA.hy926—3.3	[14]
**22**	–	+	–	GBM—0.87 GBM2—1.43 GBM3—1.45 GBM4—1.26 GBM5—0.92 GBM6—2.32 U373 MG—>5 U87 MG—0.38 U251 MG—0.33	[15]
**23**	–	+	–	Not reported	[16]
**24**	–	+	–	K562	[17]
**25**	–	–	–	Not reported	[18]
**26**	–	+	–	DLD-1—5.063 ± 0.09	[19]
**27**	–	+	–	DLD-1—5.101 ± 0.11	[19]
**28**	–	+	–	DLD-1—5.064 ± 0.12	[19]
**29**	–	+	–	MCF-7—1.5 ± 0.7	[20]
**30**	–	+	–	MDA-MB-231—5.37 MDA-MB-231—2.67 Doxorubicin-resistant MDA-MB-231—5.70	[21]
**31**	–	+	–	Not reported	
**32**	–	+	–	22RV1 cells 48 h—8.791 72 h—8.516	[22]
**33**	+	–	–	Not reported	[23]
**34**	+	–	–	Not reported	[23]
**35**	+	–	–	1.92 (%)	[23]
**36**	–	+	–	SUM149—11.20 MDA-MB-231—18.00	[24]
**37**	–	+	–	MDA-MB-231—0.52	[24]
**38**	–	+	–	MCF-7—73.4	[24]
**39**	–	+	–	MDA-MB-231—EC50—0.42	[24]
**40**	–	+	–	MDA-MB-231—EC50—0.78	[24]
**41**	–	+	–	MCF-7	[24]
**42**	–	+	–	SUM149—13.50 MDA-MB-231—15.00	[24]
**43**	–	+	–	MCF-7—13.10 MCF-7R—12.00	[24]
**44**	–	+	–	MCF-7—2.56 MDA-MB-231—3.37	[24]
**45**	–	+	–	MCF-7—34.99	[24]
**46**	–	+	–	MCF-7—5.80	[24]

## Abbreviations

1H NMR	Proton nuclear magnetic resonance
5-FU	5-fluorouracil
5-LOX	5-Lipoxygenase
ACR	American College of Rheumatology
ADAMTS5	A disintegrin and metalloproteinase with thrombospondin motifs 5
AST	Aspartate transaminase
ALR2	Aldose Reductase 2
Arg-1	Arginase deficiency-1
Bcap-37	Human breast cancer cell line
BDC	CBA-iR: bis-demethylcurcumin
BDMC	bis-dimethoxycurcumin
C-SDs	Curcumin solid dispersions
CRP	C reactive protein
CD4+	cluster of differentiation 4
COX-1	Cyclooxygenase-1
COX-2	Cyclooxygenase-2
COMP	Cartilage Oligomeric Matrix Protein
DPPH	2,2-diphenyl-1-picrylhydrazyl
DAS	Disease activity score
DiMc	Dimethoxycurcumin
HPLC–MS	High-performance liquid chromatography-mass spectrometry
TAC	Total antioxidant capacity
MDA	Malondialdehyde
VO2 max	Maximum oxygen uptake
ROS	Reactive oxygen species
RA	Rheumatoid arthritis
NF-κB	Nuclear factor kappa-light-chain-enhancer of activated B cells
MAPK	Mitogen-activated protein kinase
MGC-803	Human gastric cancer cell line
PC3	Human prostate cancer cell line
Po bid	orally, twice daily
NIH3T3	Normal cell line
TNF-*α*	Tumor necrosis factor-alpha
MCP-1	Monocyte chemoattractant protein-1
TGF-*β*	Transforming growth factor-*β*
VAS	Visual Analogue Score
cd8+ t cells	Cytotoxic T cells
KOA	Knee osteoarthritis
IL-4	Interleukin-4
IL-6	Interleukin-6
IL-10	Interleukin-10
IL-15	Interleukin-15
IL-17	Interleukin-17
IL-22	Interleukin-22
IL-23	Interleukin-23
IL-27	Interleukin-27
IL-1*β*	Interleukin-1-beta
IL-1R	Interleukin-1 receptor
IL-12	Interleukin 12
VAS/WOMAC	Visual analogue scales for global osteoarthritis pain and the Western Ontario and McMaster University
PGE2	Prostaglandin E2
P53	tumor protein
OA	Osteoarthritis
SpO2	Saturation of peripheral oxygen
O2	Oxygen molecule
TBUT	Tear break-up time
SPEED	Sepsis patient evaluation emergency department
OSDI	Ocular Surface Disease Index
MMP-1	Matrix metalloproteinases-1
MMP-3	Matrix metalloproteinases-3
MMP-9	Matrix metalloproteinases-9
MMP-13	Matrix metalloproteinases-13
TBARS	Thiobarbituric acid reactive substances
GI	Gastrointestinal
TLR-4	Toll-like receptor 4
iNOS	inducible nitric oxide synthase
NO	Nitric oxide
p65	REL-associated protein involved in NF-κB heterodimer formation, nuclear translocation and activation
PGES	Prostaglandin E synthase
PLA2	Phospholipase A2
LOX	Lipoxygenases
mPGES-1	Microsomal prostaglandin E synthase-1
MPM	Mouse primary peritoneal macrophage
SD	Sprague–Dawley rat
DCs	JAWS II dendritic cells
GUSB	*β*-Glucuronidase
Nrf2	Nuclear factor erythroid 2–related factor 2
NGUC	Next Generation Ultrasol Curcumin
MD2	Myeloid differentiation factor 2
HU-VECs	Human Umbilical Vein Endothelial Cells
PPAR-γ	Peroxisome proliferator-activated receptor gamma
NLRP3	NLR family pyrin domain containing 3
VCAM-1	Vascular cell adhesion molecule 1
ICAM-1	Intercellular Adhesion Molecule 1
